# Landscapes of synchronous multiple primary cancers detected by next‐generation sequencing

**DOI:** 10.1002/2211-5463.13491

**Published:** 2022-10-02

**Authors:** Yiru Kong, Jing Li, Hao Lin, Xiaohua Liang, Xinli Zhou

**Affiliations:** ^1^ Department of Oncology Huashan Hospital Fudan University Shanghai China; ^2^ Department of Oncology Shanghai Medical College Fudan University China

**Keywords:** ALK, next‐generation sequencing, PKB signaling pathway, SNVs, synchronous primary cancer

## Abstract

An increase in the detection rate of multiple primary cancers has been accompanied with declining cancer death rates over the past few decades. However, synchronous multiple primary tumors have gradually increased, and the molecular mechanisms involved in the synchronous occurrence of multiple primary cancers of different origins are unclear. To investigate these mechanisms, we sequenced cancer tissues by FoundationOne CDx. Data were annotated with annovar, and we then performed pathway enrichment analysis. A total of 109 genes that were mutated in all samples were clustered into different diseases, biological processes, and molecular functions. GO and KEGG analyses indicated that the P53 and PKB signaling pathways may be relevant to the occurrence of synchronous multiple primary cancers. In summary, patients with a concordance of mutations in pathogenetic genes may have a higher risk of developing a second cancer. Our research may provide a basis for the development of individualized treatments for synchronous multiple primary cancers.

## Graphical abstract

The molecular mechanisms involved in the synchronous occurrence of multiple primary cancers of different origins are unclear. To investigate these mechanisms, we subjected 18 samples from nine patients with synchronous primary tumors to next‐generation sequencing. We identified a total of 109 genes with mutation sites. The PKB and P53 signaling pathways were found to be related to the progression of synchronous primary tumors.

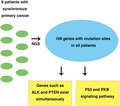

**Abbreviations**
IACR/IARC: International Agency for Research on Cancer
NGS: next‐generation sequencing
SNVs: somatic nucleotide variants
TSAs: tumor‐specific antigens

Multiple primary cancers are defined as more than one synchronous or metachronous cancer in the same individual. Although scientists have proposed many different definitions of multiple primary cancers over the past few years, the most commonly used definitions come from the International Association of Cancer Registries and International Agency for Research on Cancer (IACR/IARC) [1]. According to the IARC, synchronous multiple primary cancers are considered to occur at the same time if tumors at different sites appear within 6 months of the time of diagnosis (more than 6 months is considered to occur at different times) [2]. Due to improvements in early detection and treatment, cancer mortality has been declining in the past 20 years, and the detection rate of multiple primary cancers has gradually increased [3]. According to epidemiological studies by Professor Stefano Rosso, the incidence of multiple primary cancers in registered survivors of cancer patients is between 0.4% and 12.9% [4]. There have been studies of multiple system lesions caused by mutations in a single representative gene. Despite this, research on the mechanism of the occurrence and development of multiple primary cancers is still quite preliminary at present. Moreover, a lack of understanding regarding multiple primary cancers may limit the timely clinical detection, early intervention, and primary prevention of such patients. There is an increased need to study the driving genes and pathogenesis of multiple primary cancers.

We performed next‐generation sequencing (NGS) on nine patients with synchronous primary cancers, including lung cancer, gastric carcinoma, prostate cancer, renal clear cell carcinoma, and breast cancer. We subsequently identified somatic nucleotide variants (SNVs) and the synchronous primary cancer‐associated driver genes. Our research provides insights into the molecular mechanisms of synchronous primary cancer and provides new ideas and directions for future research.

## Materials and methods

### Patients and corresponding samples

Clinical data of all nine cases and formalin‐fixed, paraffin‐embedded cancer tissues were collected from Huashan Hospital from December 2018 to October 2020. The age of the nine patients ranged from 33 to 76. The median age of all the patients was 65. The detailed information of these patients is summarized in Tables 1 and S1. Written informed consent was signed before the research started. This study was authorized by the Ethics Committee of Huashan Hospital Fudan University. The institutional approval number is 2021‐922. All procedures were carried out in accordance with the ethical code of the Declaration of Helsinki.

**Table 1:** Clinical and histopathological characteristics. TNM staging is for malignant tumors. CT, chemotherapy; M, distant metastasis; N, lymph node involvement; ND, not determined; T, tumor size and local invasion range.

Number	Age	Sex	Histological type	Diagnostic interval time	Clinical stage	Treatment
P1	58	Female	Stomach adenocarcinoma	1 month	Ib	CT + Surgery + CT
			Pancreas ductal adenocarcinoma		Ia	CT + Surgery + CT
P2	69	Male	Lung adenocarcinoma	2 months	Ib	Surgery
			Kidney clear cell carcinoma		I	Surgery
P3	74	Male	Lung squamous cell carcinoma	1 month	IV	Radiotherapy + Targeted therapy
			Stomach adenocarcinoma		IV	CT
P4	70	Female	Lung adenocarcinoma	2 months	I	Surgery + CT
			Breast carcinoma		IIa	Surgery + CT + Targeted therapy
P5	61	Male	Prostate acinar adenocarcinoma	1 month	IVa	Endocrine therapy
			Stomach adenocarcinoma		IV	Targeted therapy + CT
P6	56	Male	Kidney clear cell carcinoma	6 months	I	Surgery
			Non–small cell lung carcinoma		Ib	Surgery + CT
P7	76	Male	Colon adenocarcinoma	1 month	IIIb	Surgery + CT
			Lung squamous cell carcinoma		ND	ND
P8	74	Female	Lung adenocarcinoma	3 months	IIIb	Surgery
			Colon adenocarcinoma		Ia	Surgery + CT
P9	33	Female	Stomach adenocarcinoma	1 month	IV	CT
			Thyroid papillary carcinoma		I	Surgery

### Next‐generation sequencing

All specimens were sequenced by FoundationOne CDx (F1CDx), an FDA‐approved 324‐gene panel assay conducted by DIAN (Hangzhou Lab, Dian Diagnostic Technology Co. Ltd, Sandun, Hangzhou, Zhejiang province, China) with licensed technologies. At least 50 ng of DNA was separated from each sample and sequenced to a high and uniform coverage.

### H&E staining

The paraffin blocks were sliced into 5‐μm‐thick sections and mounted onto glass microscope slides. Subsequently, before staining with H&E, the slides were deparaffinized using xylene and a series of graded alcohols. Two pathologists who were unaware of the study examined five microscopic fields randomly selected from each slide under the microscope.

### Analysis of sequence data

All the DNA sequencing data were analyzed for SNV and indels. We used reference genome with version hg19/GRCh37 in the study. First, we used FastQC for sequencing quality control. We defined a base quality score of lower than 28 as poor quality, which should be trimmed by fastx_trimmer in fastx_toolkit (0.0.14). Then, bwa (Wellcome Trust Sanger Institute, Wellcome Trust Genome Campus, Cambridge, SA, UK) (0.7.17) was used for alignment. Calling mutation was performed with The Genome Analysis Toolkit (GATK‐4.1.9.0) and haplotypecaller, whose output format is Variant Call Format (VCF). VCF files were annotated by the annovar software (Center for Applied Genomics, Children's Hospital of Philadelphia, PA, USA) (2015‐06‐17), which outputted annotated mutation data through separate tabs. Consequently, the data were converted to mutation annotation format (MAF) by the function annovartomaf in the r package maftools. Then, we filtered the MAF data by removing “NA” in VAF, “intergenic” and “intronic” in variant, and “synonymous SNV” in ExonicFunc.refGene. Finally, we used the r package clusterprofiler for GO and KEGG analysis.

## Results

### Somatic variants revealed by next‐generation sequencing

All patients in our research were diagnosed with a second tumor less than 6 months apart. Of the nine patients, five were men. Eight patients were older than 55 years when they were first diagnosed, except patient 9, who was only 33 years old at the time of first diagnosis. The 18 specimens in this study included five specimens of lung cancer, seven specimens of gastrointestinal tumors, two specimens of prostate cancer, two specimens of renal clear cell carcinoma, one specimen of breast cancer, and one thyroid papillary carcinoma. The tumor diagnosis interval, ages at diagnosis, primary tumor presentation, and patient status are shown in Tables 1 and S1. We noticed that all the patients were primarily presented with typical associated tumor symptoms, and under most circumstances the second tumor was discovered during routine examinations. This reminds us that careful routine imaging examination is important and necessary.

**Fig. 1:**
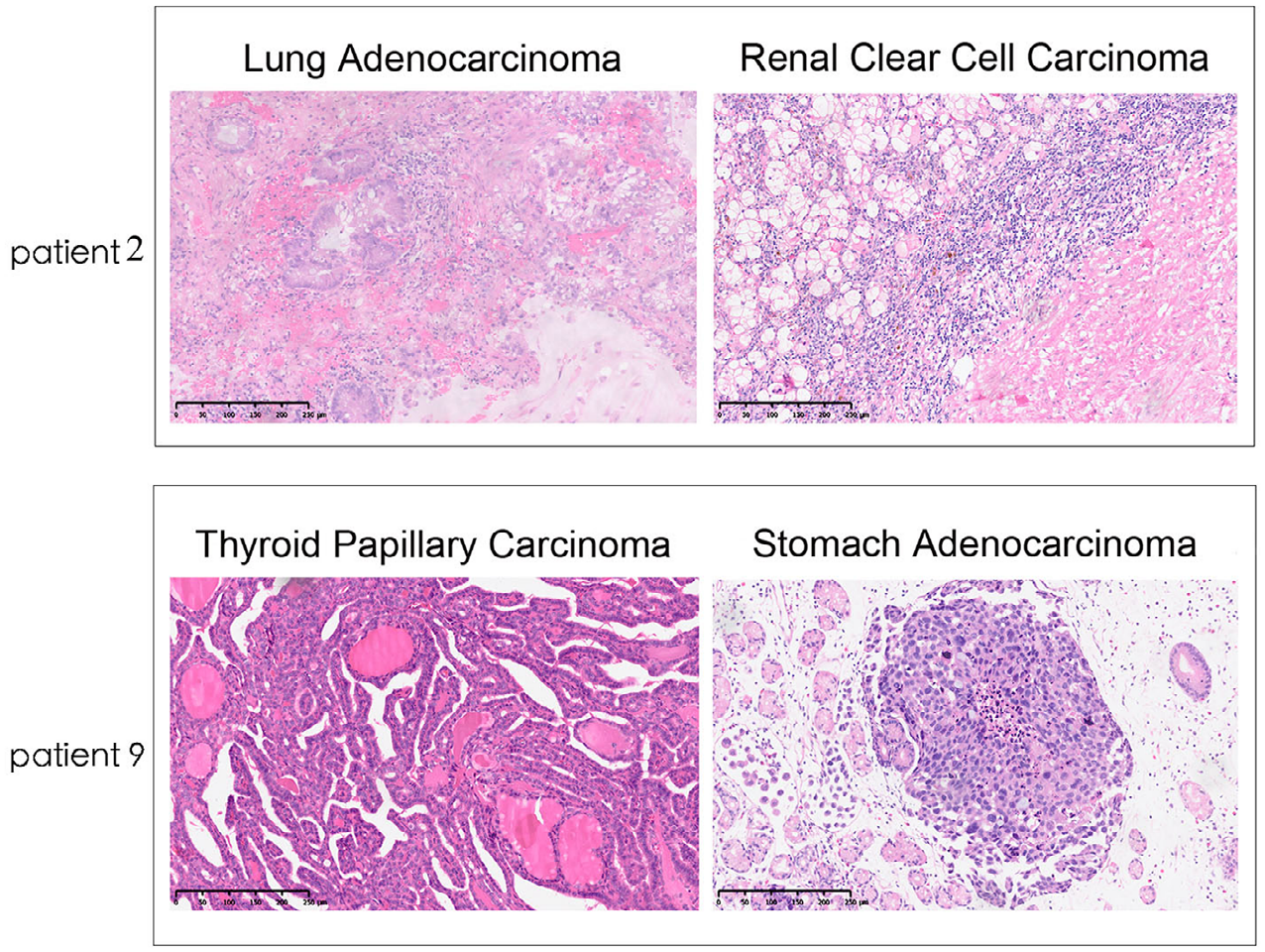
Representative images of hematoxylin and eosin staining of patient 2 and patient 9. Images of lung adenocarcinoma and renal clear cell adenocarcinoma from patient 2. Picture of thyroid papillary carcinoma and stomach adenocarcinoma (NOS) come from patient 9. Scale bar, 250 μm.

Regarding the histological type of all specimens, 11 specimens were adenocarcinoma, six specimens were carcinoma, and one specimen was not otherwise specified. Figure 1 shows representative slice pictures of patient 2 and patient 9.

**Fig. 2:**
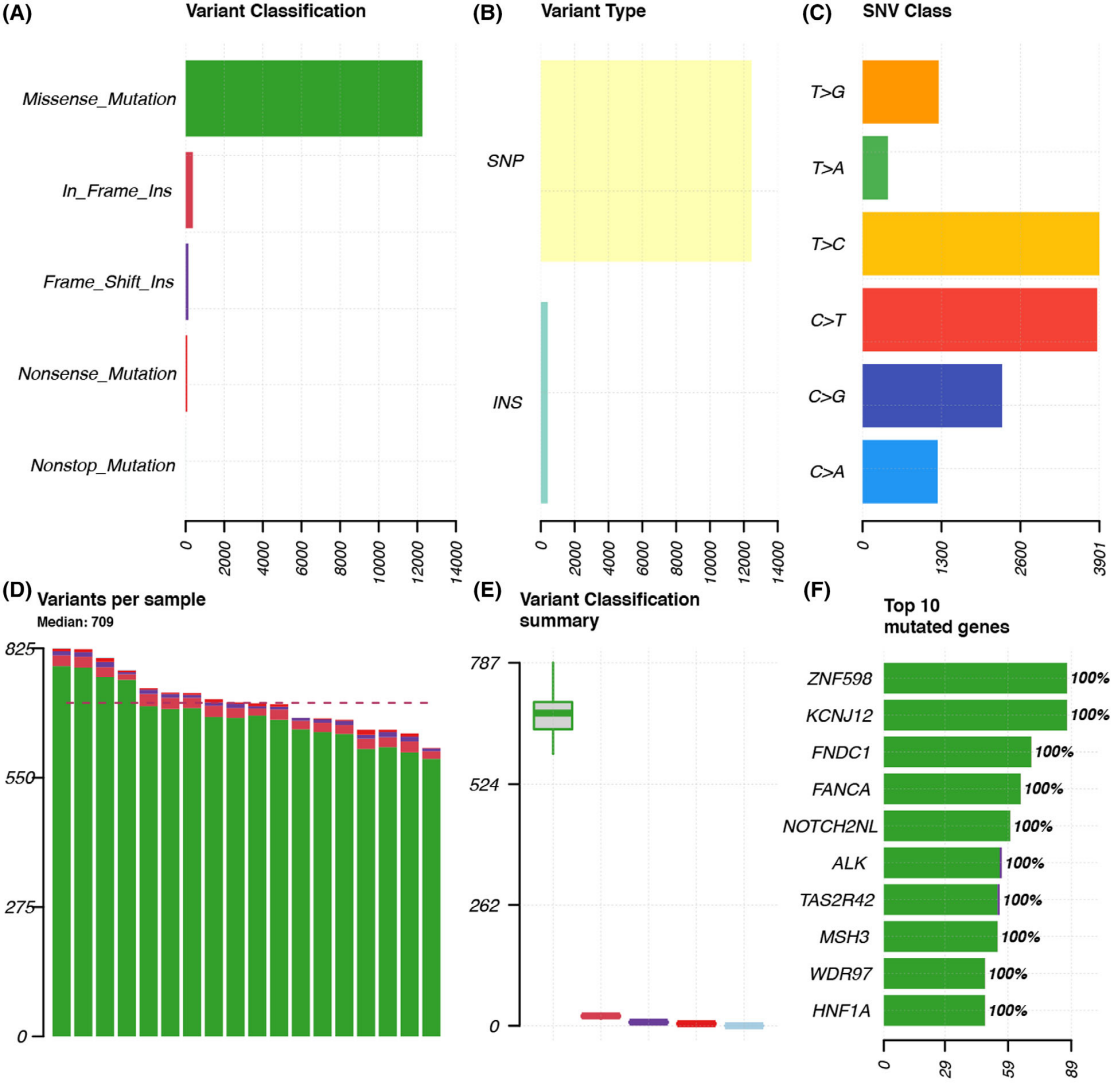
Landscape of mutations in synchronous multiple primary cancer. (A) Shows variant classification. Missense mutations are shown in green, in‐frame insertions in red, frame‐shift insertions in purple, nonsense mutations in orange, and nonstop mutations in blue. (B) Statistics of variant types. (C) Statistics of the frequency of six single‐nucleotide variants. (D) This figure shows the number of variants in each of the 18 samples. (E) Shows variant classification summary, and (E) shows the median and aggregation distribution more prominently compared to (A). (F) This figure shows the top 10 mutated genes identified from each specimen.

Technological advances have made “NGS” technology more reliable. Here, we performed a targeted sequencing panel of 324 cancer‐related genes (including ABL1, BCL2, CDH1, EGFR, etc.) at an average depth of 500× in all the specimens to identify mutations in genes that may be pivotal for synchronous primary cancer (BioProject accession number: PRJNA761143). After removing “NA” in VAF, “intergenic” and “intronic” in variants, and “synonymous SNV” in ExonicFunc.refGene, we screened out 12 861 somatic variant sites in 2320 genes. Figure 2D summarizes the heterogeneity of all samples in detail, and the sample corresponding to each bar as well as the statistical data of mutation sites in chromosomes are written in Table S2. For all the specimens, the number of medium variants per sample is 709. The top 10 mutated genes are shown in Fig. 2F. We identified five types of mutations within each primary tumor sample and found that missense mutations had the highest number of exon somatic variants in our analysis, followed by in‐frame insertions, and frame shift ranked third (Fig. 2A,E). The incidence of single‐nucleotide polymorphisms (SNPs) was the highest among the nine pairs of tumor tissue samples (Fig. 2B).

**Fig. 3:**
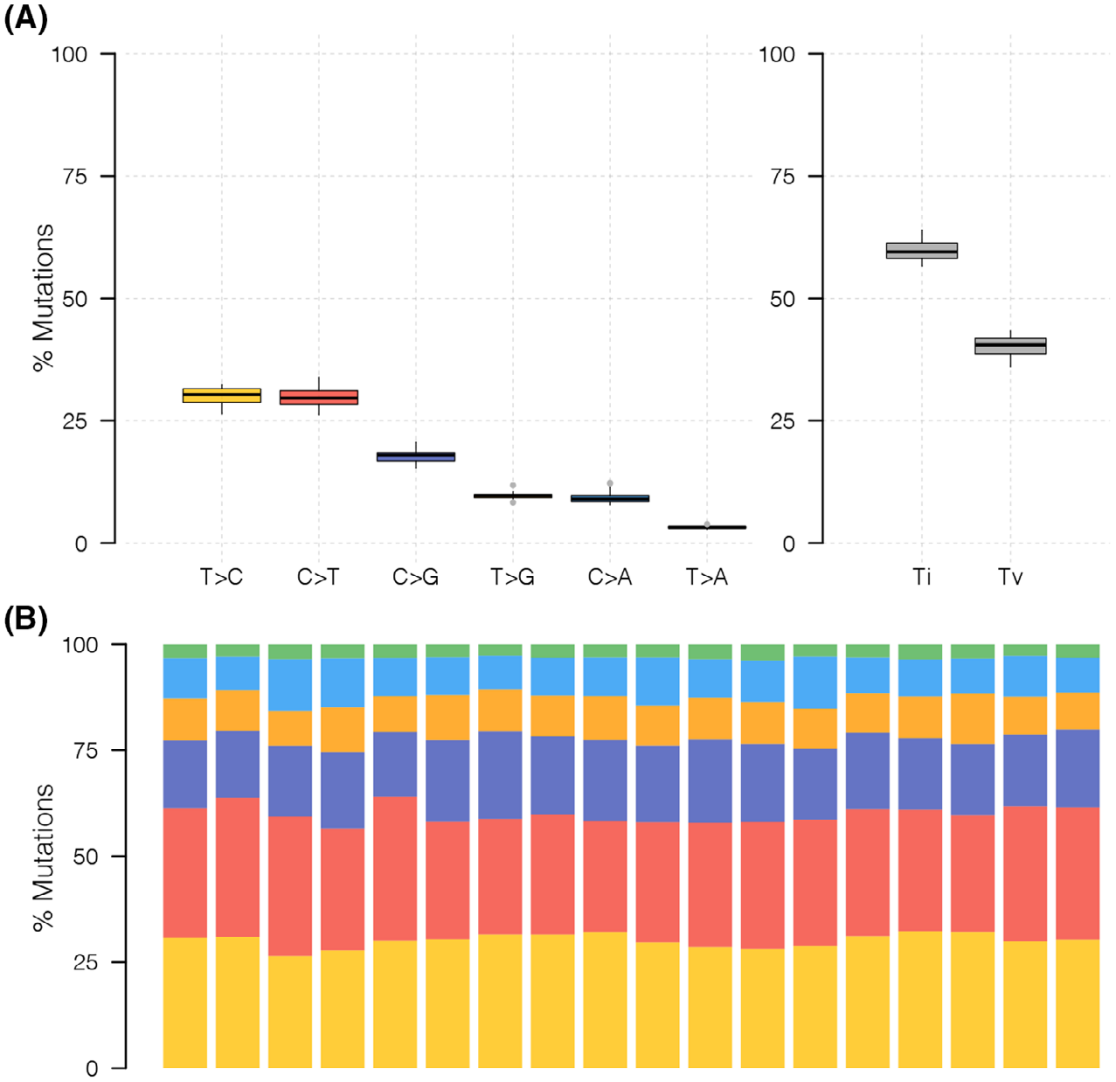
The single‐nucleotide polymorphism (SNP) landscape of all synchronous multiple primary cancers. (A) The percentage of six types of SNPs in all samples. (B) The percentage of transversion and transition in each sample.

Based on this fact, we examined the composition of six possible base‐pair substitutions and found out that C > T and T > C accounted for nearly 50% of all SNP types, which was the two highest proportions of all transition types among the nine cases (Fig. 2C). The SNP landscape of all patients with synchronous primary cancer is shown in Fig. 3.

### Distribution of single‐nucleotide polymorphisms on chromosomes

**Fig. 4:**
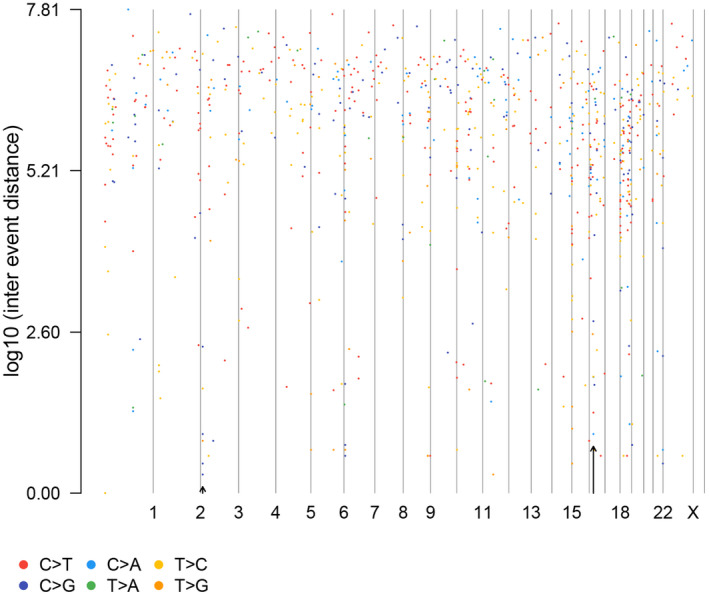
The single‐nucleotide polymorphism distribution of 23 pairs of chromosomes in the lung adenocarcinoma sample from patient 2.

Of all the samples, the lung adenocarcinoma sample in patient 2 had the highest 825 variations. Then, we visualized the SNP distribution of 23 pairs of chromosomes in this sample with the highest mutation frequency. The most notable mutational variants can be observed on chromosomes 1, 17, and 19, and the distribution of variants on chromosomes 14, 18, and 21 is relatively minimal. Figure 4 shows the distribution of mutations in the genome of this sample, where different colored dots represent different single‐nucleotide variants. This patient had lung adenocarcinoma and kidney clear cell carcinoma synchronously. Among the mutation types in this sample, missense mutations accounted for nearly 80%. Remarkably, among this specimen, there were a total of 825 mutation sites in 684 mutated genes, of which only one KRAS gene (non–small cell lung cancer OMIM ID: 211980) was certified as pathogenic by ClinVar. Moreover, while chromosomes with the least distribution of SNPs were basically different in each sample, we noticed that the SNPs of chromosomes 1, 17, and 19 had the highest frequency of occurrence in all samples except the lung adenocarcinoma sample of patient 4 (chromos 1, 9, and 19; Table S2).

### Mutation landscape of all patients after their own overlap

**Fig. 5:**
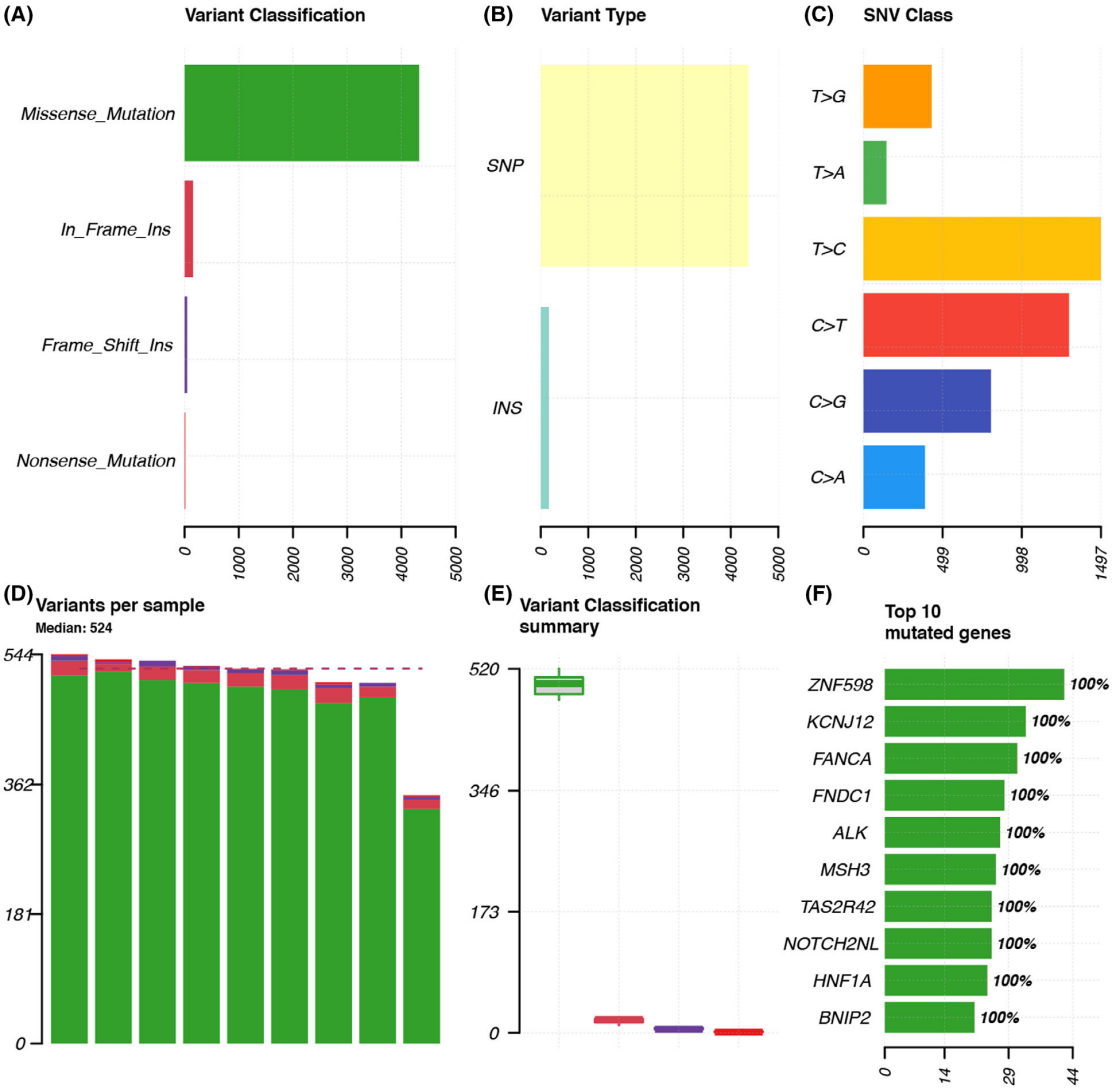
Landscape of mutations in synchronous multiple primary cancer. (A) Shows variant classification. Missense mutations are shown in green, in‐frame insertions in red, frame‐shift insertions in purple, nonsense mutations in orange, and nonstop mutations in blue. (B) Statistics of variant types. (C) Statistics on the frequency of six single‐nucleotide variants. (D) This figure shows the number of variants in nine patients. (E) Shows variant classification summary, and (E) Shows the median and aggregation distribution more prominently compared with (A). (F) This figure shows the most altered cancer‐related genes identified from each specimen.

We combined two samples from each of the nine patients together to take the intersection and searched for the common mutation of the two samples from the same patient. The observed common mutations in two samples from all patients are summarized in Fig. 5D. For all the specimens, the median number of variants per sample was 524. The most common type of mutation was a SNP (Fig. 5B). The highest proportion of all transition types in the SNV class was T > C (Fig. 5C). Moreover, we found that missense‐mutations occurred most frequently in our analysis, which is consistent with the results obtained in our previous analysis (Fig. 5A,E). Figure 5F shows the top 10 genes with the highest number of mutation sites in all patients.

### A total of 109 genes with mutation sites in all 18 samples were identified

After analyzing all the mutation sites, we identified 109 genes with mutation sites in each of the samples (Table S3). The variant allele frequency of the mutations identified in each gene in all samples is shown in Table S4. We noticed that only FGFR4 (cancer progression and tumor cell motility, OMIM ID: 134935) is classified as pathogenic in ClinVar. FGFR4 has been identified as deleterious by prediction algorithms, including SIFT, Polyphen2, and FATHMM. It is plausible that mutations in many different genes can work together to drive the development of cancers, while our sample size is relatively small, and more large scale, and comprehensive genetic research needs to be carried out. To further mine the genes that were mutated in all the patients, we performed cluster analysis to determine the molecular functions (MFs) of these genes, as well as their relations with diseases.

### Functional prediction and bioinformatic analysis of synchronous primary cancer‐related genes

**Fig. 6:**
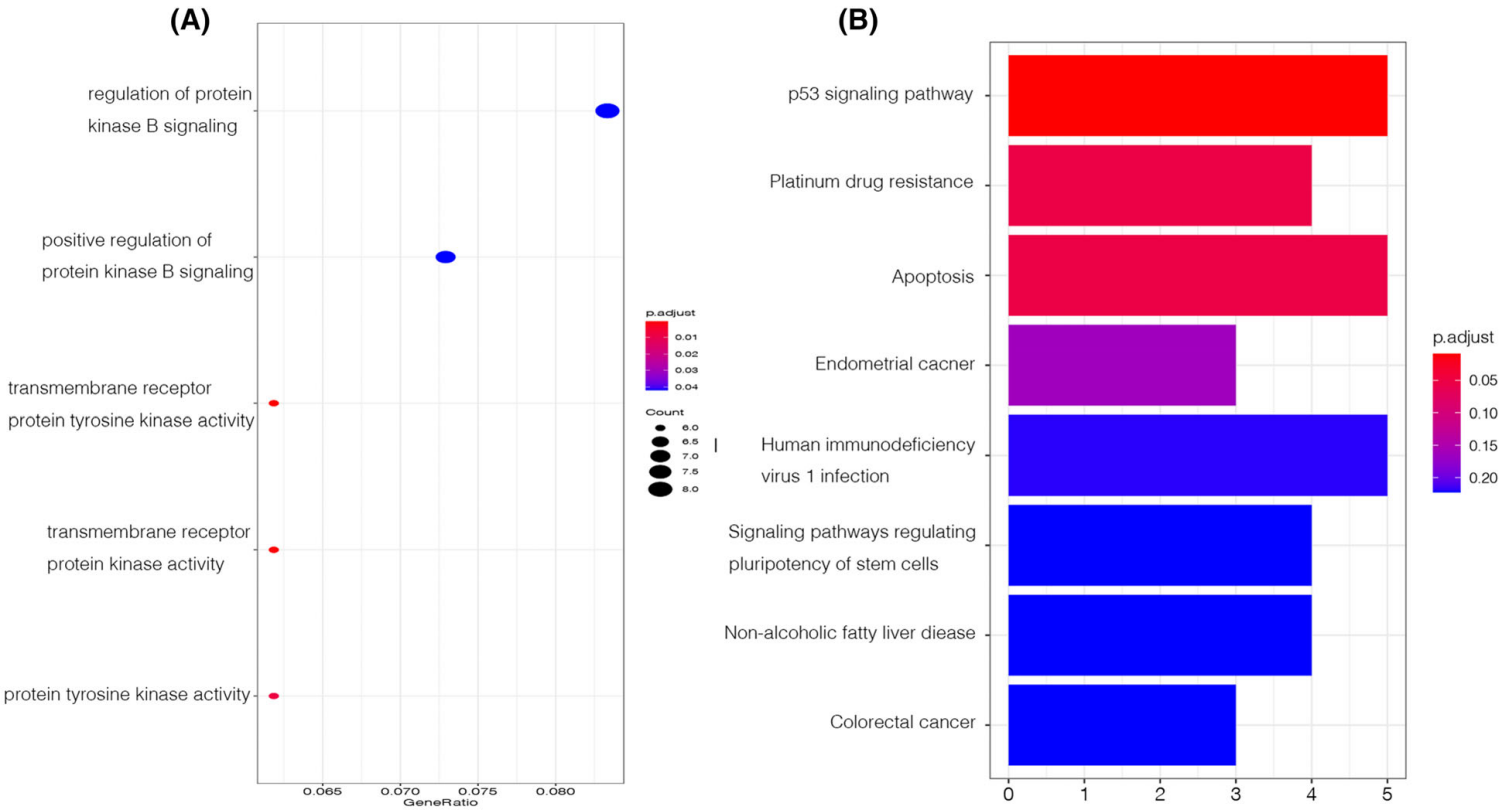
Functional prediction and bioinformatic analysis of synchronous multiple primary cancer‐related genes. (A) Shows the GO‐MF, GO‐BP, and GO‐CC results, which were ranked by GeneRatio. Dots indicate the number of genes, and the colors indicate the *P* values. (B) Was used to describe the KEGG analysis including molecular interactions, reactions, and relational networks of the 109 genes identified from all samples.

We used the r package clusterprofiler for pathway enrichment analysis to further examine the cellular components, MFs, and biological processes (BPs). The shared pathogenic genes, which identified from synchronous primary cancer (Tables S5 and S6) were categorized according to the functional gene sets in the Gene Ontology (GO) and Kyoto Encyclopedia of Genes and Genomes (KEGG) databases. We found that the results of GO signaling pathway enrichment analysis were associated with BP and MF, including positive regulation of protein kinase B (PKB) signaling (AXL, CALCR, FGFR4, HCLS1, IRS2, MST1R, TEK, *P* = 0.0420864) and regulation of PKB signaling (AXL, CALCR, FGFR4, HCLS1, IRS2, MST1R, TEK, PTEN, *P* = 0.0420864). Six genes, ALK, AXL, FGFR4, FLT3, MST1R, and TEK, were clustered into three MFs involving transmembrane receptor protein tyrosine kinase activity (*P* = 0.0002543), transmembrane receptor protein kinase activity (*P* = 0.0004972), and protein tyrosine kinase activity(*P* = 0.0065611; Fig. 6A). We further conducted pathway enrichment analysis by the r package clusterprofiler, and the selected genes were categorized according to the functional gene sets in the GO and KEGG databases. Relational signaling pathways and diseases of the 109 genes identified from all the 18 samples (Fig. 6B). According to the results of KEGG signaling pathway enrichment, 109 genes were clustered into different signaling pathways, among which the P53 signaling pathway was the most reliable (ATM, CASP8, CASP9, CHEK1, PTEN, *P* = 0.00893017). In addition, four genes (ATM, CASP8, CASP9, and MSH3) were related to the tolerance to platinum drugs, which may be associated with the occurrence of synchronous multiple primary cancer. In addition, the number of genes associated with Alzheimer's disease was the highest but with low confidence (*P* = 0.237381026).

## Discussion

With advances in medical technology, many tumors are found at very early stages, leading to an increase in the number of cancer patients alive. One retrospective, single‐institution study with respect to esophageal cancer and multiple primary cancers found that the overall survival of patients with synchronous cancer is significantly shorter than that of patients without multiple primary cancers [5]. In some cases, multiple primary cancers have obvious characteristics, and the commonality of many such cases proves that they are almost certainly of genetic origin, which is called a syndrome. These conditions include hereditary breast ovarian [6]; Cowden syndrome, which brings about malignancies including breast, thyroid, and endometrial [7]; and colon and endometrial cancer: Lynch syndrome [8]. Nine patients in this research are found with no evidence of this hereditary mutation. Eighteen specimens used in this study included gastrointestinal cancer, lung cancer, urinary tumors, and breast cancer, covering several tumors with the highest incidence and highest proportion of mortality. However, because cases of synchronous multiple primary cancers are uncommon, the number we obtained may be insufficient.

According to IACR/IARC, synchronous multiple primary cancers are considered to occur at the same time if tumors at different sites appear within 6 months of the time of diagnosis (metachronous if more than 6 months). Despite differences in diagnosis interval, metachronous multiple primary cancers may also be associated with treatment (including radiotherapy and alkylating agents). Nonetheless, the molecular mechanism involved in the synchronous occurrence of multiple primary tumors of different origins is unclear. Literature describing the molecular mechanisms of multiple primary cancers is very limited. Hu et al.'s [9] study collected the clinicopathological characteristics of 400 female patients clinically diagnosed with double primary lung cancer and breast cancer and determined that hormone receptor expression correlates with EGFR gene mutation. Because the genetic heterogeneity of multiple primary cancers is complicated, research on it is still frustrating. Knowing more about the pathogenesis, genetic heterogeneity, and risk factors for multiple primary cancers will enable us to help high‐risk patients with early intervention and treatment, and even prevent their occurrence.

We analyzed the 18 samples of nine patients via NGS and systematically defined many typical SNVs. Haplotypecaller in The Genome Analysis Toolkit (GATK‐4.1.9.0) was used to analyze the mutations in the samples. In our cohort, SNV was the most common type of mutation. Currently, there are many studies on tumor‐specific antigens (TSAs), and we found that TSAs are basically from SNVs [10]. These single nucleotide variants may have increased the burden of pathogenic mutations in the genes we detected with higher mutation rates. From these nine patients, it is likely that synchronous cancer presentation may be an age‐driven phenomenon. Some mutational processes carry out continuously, while normally functioning cells may deal with a mutation load that is proportional to the age of the person, with more mutations present in older individuals [11]. Although some somatic mutations are related to the age of the patient at cancer diagnosis, distinct mutational signatures are present in different cancer classes. The structural variant signatures show considerable heterogeneity across tumor types and patients with a given tumor type [12]. Moreover, the mutational characteristics of different tumor tissues provide new insights into distinguishing variant subgroups of etiology and providing therapeutic benefits [13, 14]. However, due to our limited sample size, the association between SNPs and antitumor therapy needs more research for in‐depth mining.

In addition to the definition of mutation sites in the synchronous multiple primary samples, we also classified mutated genes with various diseases, BPs, and MFs through cluster analysis. Notably, mutations in ALK and PTEN are concordant in a range of tumor types, including lung adenocarcinoma, endometrial cancer, colorectal cancer, and triple‐negative breast cancer [15, 16, 17, 18, 19]. Previously, a study examining clinically relevant mutations in 91 different malignancies also found mutation sites in anaplastic lymphoma kinase (ALK) and PTEN [20]. Genetic alterations of ALK, including point mutations, deletions, and rearrangements, have been described in a range of tumor types and tumorigenesis [21]. ALK plays an important role in the occurrence and metastasis of non–small cell lung cancer, malignant peritoneal mesothelioma, gynecologic clear cell carcinoma, melanoma, etc. [22, 23, 24, 25]. As written in the previous part of this article, it was initially reported that approximately 80% of patients with Cowden syndrome had an identifiable germline PTEN mutation [7]. PTEN has also been reported to be relevant to many different types of tumors, including prostate cancer, breast cancer, and hepatocellular carcinoma [26, 27, 28]. Because our cases are mainly lung cancer, digestive system tumor, and breast cancer, it is possible that the patient has a higher risk of developing a second cancer when pathological mutations in ALK and PTEN exist simultaneously.

GO and KEGG analyses were used to cluster the genes into different MFs and BPs, and the most notable results were the two signaling pathways: PKB signaling pathway and P53 signaling pathway. PKB, also known as, the serine/threonine kinase Akt, is a central node in cell signaling downstream of growth factors, cytokines, and other cellular stimuli. Aberrant loss or gain of Akt activation underlies the pathophysiological properties of a variety of complex diseases, including type 2 diabetes and cancer [29]. P53 is one of the most well‐studied tumor suppressors. It is mutated or missing in half of all cancers, and the dysregulation of the p53 signaling pathway present in almost all tumors [30, 31].

## Conclusion

In aggregate, these data show that the pathogenicity of genes with a high mutation rate may come from the burden of SNVs. Moreover, patients with the concordance of mutations in pathogenetic genes may have a higher risk of developing a second cancer. New therapeutic strategies are needed to predict and conquer the hazards of tumorigenesis.

## Conflict of interest

The authors declare no conflict of interest.

## Author contributions

YK was involved in data curation, methodology, writing—review and editing. JL was involved in the formal analysis, investigation, writing—review and editing. HL was involved in resources, writing—review and editing. XL was involved in the resources, writing—review and editing. XZ was involved in conceptualization, resources, writing—review and editing.

## Supporting information


**Table S1.** The detailed information of nine patients.

**Table S2.** The distributions per chromosome of all samples.

**Table S3.** The list of 109 genes with mutation sites in each of the samples.

**Table S4.** The percentage change in variant allele frequency in the 109 genes.

**Table S5.** The categorization of the 109 genes according to the functional gene sets in the Gene Ontology (GO) database.

**Table S6.** The categorization of the 109 genes according to the functional gene sets in the Kyoto Encyclopedia of Genes and Genomes (KEGG) databases.

## Data Availability

The data underlying this article are available in the SRA database at https://www.ncbi.nlm.nih.gov/sra/PRJNA761143. The data will be available from January 1, 2023.
