# Supplementary material for: Landscapes of synchronous multiple primary cancers detected by next‐generation sequencing
Source: FEBS Open Bio. 2022 Oct 2;12(11):1996–2005. doi: 10.1002/2211-5463.13491 (PMC9623518; doi:10.1002/2211-5463.13491)
Supplement: Supplementary file 1 — Table S1. The detailed information of nine patients. [file FEB4-12-1996-s004.docx]

| Number | Histological Type | Status | Primary cancer presentation |
| --- | --- | --- | --- |
| P1 | Stomach adenocarcinoma | Dead time: 2021.8 | Abdominal pain and diarrhoea |
|  | Pancreas ductal adenocarcinoma |  | Waist abdomen ache |
| P2 | Lung adenocarcinoma | Alive | Abnormal chest radiographs were found during routine examination |
|  | Kidney clear cell carcinoma |  | Lower back pain |
| P3 | Lung squamous cell carcinoma | Dead time: 2021.5 | Left limb weakness （symptoms of brain metastases） |
|  | Stomach adenocarcinoma |  | lesion was found on gastroscopy |
| P4 | Lung adenocarcinoma | Alive | Lesion was found in routine radiography examination |
|  | Breast carcinoma |  | Right breast mass |
| P5 | Prostate acinar adenocarcinoma | Alive | Urgent urination, nocturnal urine increase |
|  | Stomach adenocarcinoma |  | Acid reflux, belching, poor appetite |
| P6 | Kidney clear cell carcinoma | Alive | Lesion was found in routine radiography examination |
|  | Non-small cell lung carcinoma |  | Lesion was found in routine radiography examination |
| P7 | Colon adenocarcinoma | Alive | Chronic abdominal pain |
|  | Lung squamous cell carcinoma |  | Lesion was found in routine radiography examination |
| P8 | Lung adenocarcinoma | Alive | Lesion was found in routine radiography examination |
|  | Colon adenocarcinoma |  | Acute abdominal pain |
| P9 | Stomach adenocarcinoma | Dead time: 2022.7.2 | Chronic abdominal pain |
|  | Thyroid papillary carcinoma |  | Lesion was found in routine radiography examination |

Table S1
